# Willingness to Receive COVID-19 Booster Dose Using the Health Belief Model (HBM) Among University Students: Cross-Sectional Study

**DOI:** 10.3390/jcm13247610

**Published:** 2024-12-13

**Authors:** Yousef Saeed Alqarni, Fahad T. Alsulami, Farah Kais Alhomoud, Faten Alhomoud, Dhafer Alshayban, Khalid A. Alamer, Bashayer Alshehail, Mohammed M. Alsultan, Ahmed A. Alanazi, Majed A. Algarni, Haifa Abdulrahman Fadil

**Affiliations:** 1 Pharmacy Practice Department, College of Clinical Pharmacy, Imam Abdulrahman Bin Faisal University-Dammam-Eastern Province-Kingdom of Saudi Arabia, P.O. Box 1982, Dammam 31441, Saudi Arabia; fkalhomoud@iau.edu.sa (F.K.A.); falhomoud@iau.edu.sa (F.A.); dmalshayban@iau.edu.sa (D.A.); kaalamer@iau.edu.sa (K.A.A.); bmalshehail@iau.edu.sa (B.A.); mmaalsultan@iau.edu.sa (M.M.A.); aasalanazi@iau.edu.sa (A.A.A.); 2 Clinical Pharmacy Department, College of Pharmacy, Taif University, P.O. Box 11099, Taif 21944, Saudi Arabia; f.alsulami@tu.edu.sa (F.T.A.); m.alqarni@tu.edu.sa (M.A.A.); 3 Department of Pharmacy practice, Faculty of Pharmacy, Taibah University, Almadinah Almunawarah 30078, Saudi Arabia; hfadil@taibahu.edu.sa

**Keywords:** attitudes, COVID-19, health knowledge, Health Belief Model (HBM), vaccination, willingness

## Abstract

**Background/Objectives:** COVID-19 has significantly impacted lives, and data show that receiving a booster vaccination has been demonstrated to lower the spread of COVID-19 and reduce the severity of the risk of infection. The Saudi government has actively promoted booster dose vaccines among university students who can spread the virus to older populations, especially in high-density environments, where the risk of virus transmission and spread is elevated. This study focuses on the acceptance of COVID-19 booster shots among students at Imam Abdulrahman bin Faisal University. The study assessed students’ willingness to receive a COVID-19 booster dose and the factors influencing their decision. **Methods:** A descriptive, cross-sectional study design using an online self-administered survey was conducted among medical and non-medical students at Imam Abdulrahman bin Faisal University. A convenience sampling technique was used to recruit participants via email and social media platforms (WhatsApp version 2.3). Quantitative analysis was performed using IBM SPSS version 28.0. using descriptive statistics. Logistic regression analysis was used to predict factors affecting COVID-19 booster dose acceptance and hesitancy. **Results:** Among 315 respondents, 171 (54.3%) were males, and 144 (45.7%) were females. All the respondents fell in the 18–25 years age group. About 173 (54.9%) respondents were from health-related colleges. Overall, 24.44% (77/315) agreed to get a COVID-19 vaccine booster dose. However, 77.14% (243/315) were confident of getting the vaccine whenever they wanted. About 48.88% (154/315) of respondents considered COVID-19 a serious severe infection, while 14.06% (46/315) of respondents were concerned about the probability of receiving COVID-19 immunization (World Health Organization, 2021). **Conclusions:** The study revealed that students were not accepting COVID-19 booster doses, highlighting the need for awareness campaigns to dispel myths and improve vaccination rates.

## 1. Background

COVID-19, also known as coronavirus disease, is a severe respiratory illness first reported in Wuhan, China, in December 2019. It quickly spread globally, with 761,402,282 cases and 6,886,364 deaths recorded by March 2023, affecting 224 countries [1]. SARS-CoV-2 virus belongs to the Corona-viridae family and is an RNA-encapsulated virus. It shares 80% genetic similarity with SARS-CoV and 50% with MERS-CoV, which caused earlier pandemics in 2002–2003 and 2012, respectively [1,2]. Due to the rapid spread of COVID-19, the WHO implemented Emergency Use Listings (EULs) for vaccines starting in December 2020 to reduce infection severity. Initial trials showed the vaccines’ short-term effectiveness, but efficacy declined over time. After one month, risk reduction was 88%, dropping to 47% after five months following the second dose [1,2]. As vaccine effectiveness wanes, booster doses are needed. However, vaccine hesitancy is a top global health issue, driven by fears of side effects, doubts about efficacy, and belief in natural immunity. In China, fear of vaccine safety hindered vaccination efforts, including booster doses [3,4]. Promoting vaccine acceptance is crucial to achieve herd immunity and lower COVID-19-related morbidity and mortality. Although data on booster doses are limited, administering them remains challenging. Campaigns emphasizing the importance of vaccination and evaluating public response are essential [5,6]. Therefore, this study aimed to assess university students’ willingness to receive a COVID-19 booster dose and identify factors influencing their decision. While younger individuals may be less likely to suffer from severe COVID-19 compared to the older population, they can spread the virus, especially due to their presence in a high-density environment where the risk of virus transmission and spread is elevated. Consequently, receiving a booster dose may help enhance immunity, offer protection against newer variants and reduce the risk of serious illness. This will also ensure that the universities continue in-person activities and classes without the disruption caused by lockdowns or outbreaks. To better understand COVID booster uptake among university students, this study measured their attitudes and behaviors.

## 2. Methods

### 2.1. Study Design

This study is a descriptive, cross-sectional survey conducted between November 2023 and May 2024. Data were gathered from Imam Abdulrahman Bin Faisal University’s medical and non-medical students. A convenience sampling technique was used to recruit participants via email and social media platforms (WhatsApp).

### 2.2. Sampling, Participant Recruitment and Sample Size

Based on the university’s student records, a convenience sample of registered medical and non-medical students at Imam Abdulrahman Bin Faisal University (IAU) was included, and the questionnaire was created using QuestionPro. Students with English communication challenges who had no internet access and did not provide consent to participate were excluded. According to the information provided by the university’s website in 2023 [7], approximately 30,768 students were registered at the university. Accordingly, we calculated the sample size using the Raosoft sample size calculator. With a 95% confidence level and a 5% margin of error, a sample size of 380 student respondents was required.

### 2.3. Data Collection and Instruments

A previously validated questionnaire was adapted to better align with the study’s objectives, with permission secured by the corresponding author. The questionnaire, administered exclusively in English, demonstrated moderate reliability (Cronbach’s alpha = 0.60) and satisfactory content validity [8,9,10]. A panel of five experienced academics in pharmacy practice evaluated the instrument for face and content validity, confirming its appropriateness without needing further modifications.

Data were collected using a structured questionnaire to assess various factors influencing individuals’ decisions regarding the COVID-19 booster dose. The questionnaire included sections on participants’ knowledge about COVID-19 and the COVID-19 vaccine, as well as constructs from the Health Belief Model (HBM), such as perceived susceptibility, severity, benefits, barriers, and cues to action. The Health Belief Model (HBM) was used as the theoretical framework to understand attitudes and behaviors regarding receiving the COVID-19 booster dose. The HBM explains that health-related decisions, such as getting vaccinated, are influenced by perceived risk, severity, benefits, barriers, and cues to action. In this study, the HBM helped explore how these factors affect individuals’ willingness to receive the booster dose [11,12,13,14]. Additionally, sociodemographic information and participants’ willingness to receive the booster dose were collected.

### 2.4. Data Analysis

Descriptive statistics were used to summarize the demographic characteristics and survey responses. Logistic regression analysis was employed to identify factors influencing the likelihood of receiving the booster dose while adjusting for potential confounders. Statistical significance was set at *p* < 0.05, and all data analysis was conducted using the Statistical Package for Social Sciences (SPSS) software version 28.0.

### 2.5. Ethics Approval

Ethical approval was obtained from the IRB of Imam Abdulrahman bin Faisal University (IRB number: IRB-2023-01-447) dated 1 November 2023.

## 3. Results

### 3.1. Participant’s Characteristics

Out of 315 previously vaccinated members contacted by Imam Abdulrahman bin Faisal University, all completed the survey. The respondents were 54.3% male (171/315) and 45.7% female (144/315), with a mean age of 20.94 ± 1.57 years (range: 18–25) [Table 1]. A significant association was observed between gender and response rate (Pearson χ^2^ = 10.659, Continuity Correction = 9.930) [Table 2]. Most respondents (54.3%, 173/315) were from health-related colleges, and the fewest (4.1%, 13/315) were from non-health-related majors (Pearson χ^2^ = 7.684, Likelihood Ratio = 7.864). The respondents were distributed across all study years (1st to 7th), with the highest proportion in the 4th year (29.2%, 92/315) and the lowest in the 7th year (1%, 3/315) [Table 1 and Table 2]. Statistical significance was also noted for college affiliation (Pearson χ^2^ = 5.852, Likelihood Ratio = 5.904).

### 3.2. Willingness and Attitude Toward Getting COVID-19 Vaccine Booster Dose

The respondents exhibited diverse attitudes toward receiving the COVID-19 booster dose. Of the 315 participants, 54.6% (172/315) demonstrated a low intention to receive the booster, with 46.2% of males and 64.6% of females falling into this category. Conversely, 45.4% (143/315) expressed a high intention, including 53.8% of males and 35.4% of females. The highest proportion of high intention (54.1%, 20/37) was observed among students from non-health-related colleges, while first-year students exhibited the most strong intention (68.4%, 13/19) to receive the booster. Detailed findings are presented in Table 3.

The survey results showed that participants had mixed attitudes towards receiving a COVID-19 booster dose. While 31.74% remained neutral, 43.79% (22.53% disagreed, 21.26% strongly disagreed) expressed reluctance, and 24.47% (15.23% agreed, 9.24% strongly agreed) were willing to receive the booster. Regarding the likelihood of getting the booster within the next six months, 54.1% were not inclined (26.98% disagreed, 25.12% strongly disagreed), and only 20% showed willingness. When asked if they thought they would contract COVID-19 without the booster, 55.87% disagreed (37.14% disagreed, 18.73% strongly disagreed), while only 20.95% (17.14% agreed, 3.81% strongly agreed) believed it could happen. Regarding concerns about contracting COVID-19, 68.57% did not express concern (28.57% disagreed, 40% strongly disagreed), and only 14.6% showed concern. On the perceived severity of COVID-19 complications, 51.43% agreed they were severe, while 20.32% disagreed. Similarly, when asked if they believed they would become ill if infected with COVID-19, 39.37% agreed, while 39.37% disagreed, with 28.25% remaining neutral.

The survey revealed varied perceptions regarding the effectiveness and side effects of the COVID-19 booster dose. When asked if the booster dose effectively prevented COVID-19 infection, 49.53% of respondents agreed or strongly agreed, 29.85% remained neutral, and 20.62% disagreed or strongly disagreed. Regarding concerns about severe side effects from the booster, 48.57% agreed or strongly agreed, 24.76% remained neutral, and 26.66% disagreed or strongly disagreed.

In regard to concerns about the booster’s efficacy, one-third of respondents expressed confidence (agreed or strongly agreed), a quarter of them were neutral, and one-third disagreed or strongly disagreed. Additionally, 65.71% of respondents believed finding a supplier or hospital with the booster was not difficult, while 20.64% remained neutral. Regarding the severity of COVID-19, 48.88% believed it to be a severe infection, 23.17% remained neutral, and 24.76% disagreed. Finally, when asked about booster dose efficacy in preventing disease, 45.71% agreed or strongly agreed, 32.06% were neutral, and 22.23% disagreed or strongly disagreed. The survey showed varied responses with regard to time and control over getting the COVID-19 booster. In total, 33.65% agreed they lacked time, 48.27% disagreed, and 18.1% were neutral. A total of 77.14% felt confident they could get the booster, 9.52% disagreed, and 13.33% were neutral. With regard to control over their decision, 69.84% agreed, 12.05% disagreed, and 18.09% were neutral. Regarding media influence, 35.24% agreed they had seen media promoting the booster, 35.24% disagreed, and 18.73% were neutral. Similarly, peer influence was also low, with 66.35% of respondents disagreeing, 17.45% agreeing, and 16.19% remaining neutral. This finding highlights the need for more peer-to-peer communication about the importance of the booster.

### 3.3. Health Belief Model (HBM) of Study Respondents

The respondents’ Health Belief Model (HBM) was divided into two categories: low intention and high intention to receive the COVID-19 vaccine. Among the 315 participants, 172 had low intentions, and 143 had high intentions. Group statistics revealed the following mean scores:

- Perceived Susceptibility: 1.94 for low intention, 2.71 for high intention (Levene’s test = 30.68, *t*-test = −7.976).
- Perceived Severity: 3.00 for low intention, 3.39 for high intention.
- Perceived Benefits: 2.88 for low intention, 3.79 for high intention.
- Perceived Barriers: 2.93 for low intention, 2.64 for high intention.
- Self-Efficacy: 3.66 for low intention, 4.09 for high intention.
- Cues to Action: 2.20 for low intention, 2.62 for high intention (Levene’s test = 3.416, *t*-test = −3.857).

Levene’s test for equality of variances and *t*-tests for equality of means was conducted for perceived benefits, severity, susceptibility, barriers, and self-efficacy, as shown in Table 4.

### 3.4. Pooled Standard Deviation Among Study Respondents

Cohen’s d, Hedges’ correction, and Glass’s delta values were calculated for key variables in the study. For the Overall Perceived Susceptibility Score, these values were 0.85994 and 1.00787. The values of the Overall Perceived Severity Score were 0.89852, 0.90068, and 0.84628. The values of the Overall Perceived Benefits Score were 0.92614, 0.92837, and 0.78796. A detailed summary of the point estimates, 95% confidence intervals, and standardizer values for all variables is provided in [Table 5].

## 4. Discussion

This study provides valuable insight into the university students’ willingness to receive a COVID-19 booster dose and identified factors influencing their decision. The introduction of COVID-19 vaccines significantly reduced mortality and hospitalizations. However, vaccine efficacy declined 20 weeks after the second dose [15], and booster doses became necessary, particularly against the Omicron variant. Several countries mandated booster doses starting in December 2021 [16], though individuals under 30 could only receive a third dose as of January 2022. The rate of booster uptake among Saudi students remains unclear, prompting this cross-sectional study at Imam Abdulrahman bin Faisal University to evaluate booster dose hesitancy and associated factors.

A total of 315 students were surveyed, 54.3% male and 45.7% female, aged 18 to 25 years. This demographic aligns with previous studies showing young men as a leading group in vaccination surveys [17,18]. While older individuals are generally more likely to receive booster doses [18], only 24.4% (77/315) of the students in this younger cohort expressed willingness to get the booster, reflecting lower uptake compared to older populations in other studies [19]. In our study, 45.4% (143/315) of students showed high intent to receive the booster, contrasting with other findings where 84% of young factory workers were willing to get a free booster [16,19]. Young adults often hesitate due to perceived low risk and concerns about vaccine safety [20].

Our findings showed that 54.6% of the university students surveyed were hesitant (46.2% of males and 64.6% of females) to receive a COVID-19 booster. According to Limbu and Huhmann’s (2023) [5] systematic review, the global average hesitancy rate for COVID-19 booster vaccinations was 30.72%. Their analysis also highlighted a notable increase in hesitancy from 2021 to 2022, with the mean rate rising from 29.46% in 2021 to 33.55% in 2022. Our study conducted between November 2023 and May 2024 suggests a continued upward trend in hesitancy, as more than half of the surveyed students expressed reluctance to receive the booster shot. This growing hesitancy among university students may reflect a broader global shift, indicating that vaccine hesitancy is not only persistent but possibly intensifying as time progresses. As a comparison to other countries, in Poland and China where COVID-19 booster hesitancy ranged from 5% to 30% [16,21], though many of these surveys were conducted before official booster approvals. An Algerian study reported similar hesitancy (23%), with one-fourth of respondents rejecting the booster [22]. In North America, the rate was 41.18%; in Europe, the rate was 34.81%; in Asia, the rate was 28.04%; and in South America, the rate was 27.6%.

A univariate regression analysis revealed a significant relationship between sociodemographic factors and booster acceptance. First-year students had a higher intent to receive the booster (68.4%) compared to seventh-year students (33.33%). The higher intent to receive the booster among first-year students compared to seventh-year students may reflect differences in risk perception and academic focus. Younger students, newly exposed to public health education, might view vaccination as a straightforward protective measure, while older students, with more clinical experience, may critically evaluate the risks and benefits or experience “vaccine fatigue” due to repeated campaigns.

In our study, 48.6% (84/173) of health-related college students showed high intent to receive a COVID-19 booster, compared to 53.8% (7/13) from arts and education colleges. However, the small sample size may explain the higher percentage among the latter. Only 35.23% of respondents felt media reports encouraged them to get the booster. Misinformation on social media, especially in developed settings, can significantly impact vaccine hesitancy [23]. Among female respondents, 64.6% (93/144) intended to get the booster, but only 35.4% expressed strong intent. This aligns with prior research showing that women are more hesitant, possibly due to concerns over menstrual cycle disruptions reported after vaccination [24]. Vaccine-related health concerns were a key factor in booster hesitancy, with 48.57% (153/315) of respondents expressing moderate to high levels of distress, similar to findings from previous studies. It is noteworthy that students from non-health-related colleges exhibited a higher intention to receive the COVID-19 booster dose. This could imply that non-health students, perhaps less exposed to medical training, may perceive themselves as more susceptible or feel a greater need for the added protection of a booster dose.

The survey revealed mixed attitudes toward the COVID-19 booster dose, with 54.6% of respondents showing a low intention to receive it. Female respondents were more likely to exhibit lower intention compared to their male counterparts. These gender differences could be attributed to a variety of factors, including social influences, perceived risks, or prior experiences with vaccination. Additionally, first-year students displayed the highest intention to receive the booster, which may reflect heightened caution or a greater willingness to follow public health recommendations in the earlier stages of their academic journey.

Vaccination rates in Saudi Arabia rose significantly in 2021, from less than 5% in July to 70% by the end of the year, driven by public awareness efforts [25,26]. Logistic regression analyses identified age, gender, study year, and social media use as key predictors of booster dose acceptance. College students undertaking health-related studies who accessed scientific literature showed greater interest in boosters than students from arts, education, and engineering who relied on social media for information. This highlights scientific sources’ importance over social media influence booster acceptance.

The data also highlighted important psychosocial factors influencing booster dose acceptance, as illustrated by the application of the Health Belief Model (HBM). Students with a high intention to receive the booster demonstrated higher levels of perceived susceptibility, severity, benefits, and self-efficacy compared to those with lower intentions. These findings align with the existing literature, where individuals with greater perceived risk and confidence in the vaccine’s benefits are more likely to accept vaccination. Conversely, perceived barriers, such as concerns about side effects, were more prevalent among those with low intention, indicating that addressing these concerns is crucial to improving booster uptake. Interestingly, despite widespread confidence in the booster’s accessibility (with 65.71% indicating that it was not difficult to find), only 36.52% expressed confidence in its efficacy. This points to a gap between perceived access and trust in the vaccine’s protective benefits, underscoring the need for more effective communication and education around the efficacy of booster doses. Another noteworthy finding is the low influence of peer and media messaging on participants’ decision-making, with only 17.45% reporting that peer influence affected their willingness to receive the booster. This suggests that peer-to-peer communication and social media campaigns promoting the booster could be underutilized, and efforts to engage students through these channels might help address vaccine hesitancy.

Overall, the study reveals that while many students understand the severity of COVID-19 complications, there remains hesitancy regarding the booster dose, particularly among females and those with lower self-efficacy. Public health initiatives aimed at increasing vaccine confidence, particularly by addressing concerns about side effects and emphasizing the benefits of the booster, are essential to improving uptake rates. Furthermore, leveraging peer networks and media campaigns could help shift attitudes in favor of booster vaccination, especially among students with lower intentions. Previous studies have shown that social media can spread misinformation, leading to lower vaccine acceptance [20,27]. In response, the World Health Organization (WHO) has produced social media content to dispel myths. Providing accurate information through reliable channels could improve booster uptake. Past research also indicates that healthcare workers sharing accurate data on social media effectively combats misinformation [22,23]. Collaboration between the public and private sectors is crucial for increasing booster acceptance and achieving herd immunity [17]. Further research combining qualitative and quantitative approaches is needed to explore booster hesitancy and improve vaccine outreach. Broader studies will help address biases in current research and adapt to the evolving COVID-19 situation.

## 5. Conclusions

In conclusion, the current study underscores the complexity of vaccine attitudes and highlights the need for targeted interventions to increase COVID-19 booster uptake, particularly in addressing perceived barriers and enhancing perceived benefits among hesitant groups. By doing so, universities and public health authorities can better support the health and safety of their student populations amidst the ongoing pandemic.

## Figures and Tables

**Table 1:** Sociodemographic characteristics of participants in current study (n = 315).

Sociodemographic Characteristics		Total (%)
Gender (%)		
Male	171 (54.3)	315(100.00)
Female	144 (45.7)	
College		
Health-related colleges	173 (54.9)	315(100.00)
Engineering college	30 (9.5)	
Sciences and Management colleges	62 (19.7)	
Arts and Education colleges	13 (4.1)	
Other colleges	37 (11.7)	
Study Year (%)		
1st year	19 (6.0)	315 (100.00)
2nd year	54 (17.1)	
3rd year	71 (22.5)	
4th year	92 (29.2)	
5th year	65 (20.6)	
6th year	11 (3.5)	
7th year	3 (1.0)	

**Table 2:** Chi-Square test analysis of gender-wise, college-wise and study-year-wise distribution of participants.

Statistical Analysis on Gender-Wise Willingness to take COVID Booster Dose					
Test Name	Value	Degrees of Freedom (df)	Asymptotic Significance (2-Sided)	Exact Sig. (2-Sided)	Exact Sig. (1-Sided)
Pearson Chi-Square	10.659 ^a^	1	0.001		
Continuity Correction ^b^	9.930	1	0.002		
Likelihood Ratio	10.746	1	0.001		
Fisher’s Exact Test				0.001	<0.001
Linear-by-Linear Association	10.625	1	0.001		
N of Valid Cases	315				
Statistical analysis on college-wise willingness to take COVID booster dose					
Pearson Chi-Square	7.684 ^c^	4	0.104		
Likelihood Ratio	7.864	4	0.097		
Linear-by-Linear Association	0.051	1	0.821		
N of Valid Cases	315				
Statistical analysis on study-year-wise willingness to take COVID booster dose					
Pearson Chi-Square	5.852 ^d^	6	0.440		
Likelihood Ratio	5.904	6	0.434		
Linear-by-Linear Association	2.301	1	0.129		
N of Valid Cases	315				

Keys. ^a^. 0 cells (0.0%) have expected count less than 5. The minimum expected count is 65.37. ^b^. Computed only for a 2 × 2 table. ^c^. 0 cells (0.0%) have expected count less than 5. The minimum expected count is 5.90. ^d^. Three cells (21.4%) have expected count less than 5. The minimum expected count is 1.36.

**Table 3:** Willingness toward getting booster dose of COVID-19.

Gender	Low Intention (%)	High Intention (%)	Total Students (%)
Male	79 (46.2)	92 (53.8)	315 (100.00)
Female	93 (64.6)	51 (35.4)	
Total	172 (54.6)	143 (45.4)	
College-wise distribution of students (%)			
Health-related colleges	89 (51.4)	84 (48.6)	315 (100.00)
Engineering college	17 (56.7)	13 (43.3)	
Sciences and Management colleges	43 (69.4)	19 (30.6)	
Arts and Education colleges	6 (46.2)	7 (53.8)	
Other colleges	17 (45.9)	20 (54.1)	
Total	172 (54.6)	143 (45.4)	
Study-year-wise distribution of students (%)			
1st year	6 (31.6)	13 (68.4)	315 (100.00)
2nd year	31 (54.7)	23 (42.6)	
3rd year	37 (52.2)	34 (47.9)	
4th year	50 (54.3)	42 (45.7)	
5th year	40 (61.5)	25 (38.5)	
6th year	6 (54.5)	5 (45.5)	
7th year	2 (66.7)	1 (33.3)	
Total students year-wise	172 (54.6)	143 (45.4)	

**Table 4:** Status of study participants for intention to get COVID-19 vaccine; results on based on low and high intention to get COVID booster vaccine.

Intention to Get COVID-19 Vaccine Categories		Group Statistics				Independent Samples Test									
		N	Mean	Std. Deviation	Std. Error Mean	Equal Variances Assumed						Equal Variances Not Assumed			
						Levene’s Test for Equality of Variances		*t*-Test for Equality of t	*t*-Test for Equality of Means			*t*-Test for Equality of t	*t*-Test for Equality of Means		
						f	Sig.	t	df	Mean /Std. Error Difference	95% CI Difference Lower/Upper	t	df	Mean Difference	95% CI Difference Lower/Upper
Overall Perceived Susceptibility Score	Low Intention	172	1.9390	0.709	0.054	30.68	<0.001	−7.976	313	−0.774	−0.965	−7.731	248.164	−0.77433	−0.97160
	High Intention	143	2.7133	1.007	0.084					0.097	−0.583			0.10016	−0.57707
Overall Perceived Severity Score	Low Intention	172	3.0019	0.939	0.071	1.436	0.232	−3.786	313	−0.385	−0.585	−3.823	310.979	−0.38501	−0.58317
	High Intention	143	3.3869	0.846	0.070					0.101	−0.184			0.10071	−0.18685
Overall Perceived Benefits Score	Low Intention	172	2.8808	1.026	0.078	8.506	0.004	−8.710	313	−0.912	−1.11	−8.921	311.084	−0.91289	−1.11425
	High Intention	143	3.7937	0.787	0.065					0.104	−0.706			0.10233	−0.71154
Overall Perceived Barries Score	Low Intention	172	2.9302	0.720	0.054	1.419	0.235	3.535	313	0.293	0.130	3.522	297.722	0.29387	0.12965
	High Intention	143	2.6364	0.751	0.062					0.083	0.457			0.08344	0.45808
Overall Self Efficacy Score	Low Intention	172	3.6570	0.955	0.072	14.78	<0.001	−4.538	313	−0.430	−0.617	−4.683	304.618	−0.43044	−0.61130
	High Intention	143	4.0874	0.669	0.055					0.094	−0.243			0.09191	−0.24957
Overall Cues to Action Score	Low Intention	172	2.1996	0.900	0.068	3.416	0.066	−3.857	313	−0.415	−0.627	−3.816	287.367	−0.41577	−0.63021
	High Intention	143	2.6154	1.011	0.084					0.107	−0.203			0.10895	−0.20134

**Table 5:** Pooled and sample standard deviation and correction factor of study participants.

		Standardizer ^a^	Point Estimate	95% Lower	95% Upper
Overall Perceived Susceptibility Score	Cohen’s d	0.85788	−0.903	−1.135	−0.669
	Hedges’ correction	0.85994	−0.900	−1.132	−0.668
	Glass’s delta	1.00787	−0.768	−1.006	−0.528
Overall Perceived Severity Score	Cohen’s d	0.89852	−0.428	−0.652	−0.204
	Hedges’ correction	0.90068	−0.427	−0.651	−0.203
	Glass’s delta	0.84628	−0.455	−0.682	−0.226
Overall Perceived Benefits Score	Cohen’s d	0.92614	−0.986	−1.220	−0.750
	Hedges’ correction	0.92837	−0.983	−1.217	−0.748
	Glass’s delta	0.78796	−1.159	−1.417	−0.898
Overall Perceived Barries Score	Cohen’s d	0.73454	0.400	0.176	0.624
	Hedges’ correction	0.73631	0.399	0.175	0.622
	Glass’s delta	0.75101	0.391	0.164	0.617
Overall Self Efficacy Score	Cohen’s d	0.83822	−0.514	−0.739	−0.288
	Hedges’ correction	0.84024	−0.512	−0.737	−0.287
	Glass’s delta	0.66955	−0.643	−0.876	−0.408
Overall Cues to Action Score	Cohen’s d	0.95254	−0.436	−0.661	−0.212
	Hedges’ correction	0.95483	−0.435	−0.659	−0.211
	Glass’s delta	1.01113	−0.411	−0.637	−0.184

Keys: ^a^. The denominator used in estimating the effect sizes. Cohen’s d uses the pooled standard deviation. Hedges’ correction uses the pooled standard deviation, plus a correction factor. Glass’s delta uses the sample standard deviation of the control group.

## Data Availability

The data supporting the findings of this study are available upon request from the corresponding author.
